# Adult and pediatric cystic nephroma: an easily misdiagnosed renal tumor

**DOI:** 10.3389/fonc.2026.1799009

**Published:** 2026-04-23

**Authors:** Guo run Zi, Da-jiang Zhang, Dong-Lin He, Jia-gui Chai, Chang-xing Ke, Run-lin Feng

**Affiliations:** 1 Department of Urology, The Second Affiliated Hospital of Kunming Medical University, Kunming, China; 2 Department of Pathology, The Second Affiliated Hospital of Kunming Medical University, Kunming, China

**Keywords:** Bosniak classification, cystic nephroma, misdiagnosis, pathological diagnosis, renal cysts, renal tumor

## Abstract

**Background:**

Cystic nephroma (CN) is a rare, benign renal tumor often misdiagnosed due to overlapping radiological features with simple renal cysts and malignant cystic neoplasms. CN primarily affects boys and adult females. This study aimed to review the clinical, imaging, and pathological characteristics of CN to improve diagnostic accuracy.

**Methods:**

A retrospective analysis was conducted on six confirmed CN cases treated at a single institution (2010–2024). Data included demographics, imaging findings (Bosniak classification), surgical approach, histopathology, and immunohistochemistry (IHC).

**Results:**

The cohort consisted of two males (one pediatric) and four females. Preoperative imaging, revealing multilocular cystic lesions with septal enhancement, led to a high misdiagnosis rate: five cases (83%) were misinterpreted as simple renal cysts (Bosniak II–III). Surgical interventions included partial nephrectomy (n=3), radical nephrectomy (n=1), and nephroureterectomy (n=1). Intraoperative frozen section analysis in one case was instrumental in confirming the CN diagnosis and guiding radical resection. Histopathology showed multilocular cysts lined by hobnail epithelium. IHC confirmed PAX-8 positivity (4/4) and ER/PR expression (3/4 and 2/4, respectively).

**Conclusion:**

CN as a rare benign renal tumor, preoperative differentiation of CN, particularly from benign simple renal cyst, remains difficult. Surgical excision with pathological verification is critical. Intraoperative frozen section analysis aids in determining the surgical approach (nephron-sparing vs. radical resection) for patients with lesions that are difficult to distinguish as benign or malignant prior to surgery. Pathological hallmarks and supporting IHC (PAX-8, ER/PR) remain the diagnostic gold standard.

## Introduction

Adult and Pediatric cystic nephroma, hereinafter referred to as cystic neoplasm (CN) is a rare benign renal tumor characterized by significant cystic components, benign pathological features, and biological behaviors. Classified as part of mixed epithelial and stromal tumor (MEST) family with MEST at opposite end of spectrum, by the 2016 World Health Organization (WHO) Classification. However, during early diagnosis, some patients often exhibit malignant imaging characteristics on imaging studies, Unlike simple renal cysts, CN imaging reveals multiple intra-cystic septa (1). Although the presence of DICER 1 mutations is well established in PCN (pediatric CN), the misdiagnosis rate of CN is notably high, primarily because the benign slow-growing renal cystic neoplasm which mimics other cystic renal lesions and has such clinical, radiological, and morphological features that causes diagnostic dilemma. And this disease is rare and there are limited reports to date (2, 3). Patients admitted to the hospital at an early stage are frequently diagnosed with renal cysts due to similar imaging manifestations. Clinicians typically perform conservative treatment or surgical intervention based on the Bosniak Classification System for renal cystic masses, and confirmed by pathological examination (4). Our study analyzes 6 cases of CN, concentrating on imaging characteristics, surgical strategies, intraoperative findings, and pathological morphology. Furthermore, the study reviews past research and case reports from the last five years, examining reasons for the frequent misdiagnosis of CN as simple renal cysts and the consequences of clinical mismanagement.

## Patients and methods

A retrospective analysis was conducted on the data of 6 patients with CN who received a pathological diagnosis and were admitted to the Department of Urology at the Second Affiliated Hospital of Kunming Medical University from January 2010 to September 2024. Complete clinical data included gender, age, tumor size, tumor location, clinical examinations, imaging data, surgical methods, postoperative pathological results, and prognostic follow-up. The diagnosis and treatment of these 6 patients were retrospectively analyzed. The study adhered to STROBE criteria of retrospective observational studies.

## Results

The data of 6 patients are presented in **Table 1**). Among them, five are adults (one male and 4 females), with ages ranging from 26 to 45 years. There was 1 child case, a 14-year-and-1-month-old boy. All 6 patients exhibit unilateral lesions. Specifically, lesions are located on the left side of the kidney in 4 cases and on the right side in 2 cases, with diameters varying from 3.2 to 11.3 cm. 2 patients have lesions at the upper pole, 2 in the middle part, and 2 at the lower pole of the kidney. In one instance, the lesion at the lower pole affects the renal pelvis and the upper part of the ureter. 2 cases are incidentally detected during physical examinations, 3 cases present with lumbar and abdominal pain, and one case has painless gross hematuria. No lesions are identified in the contralateral kidneys for all patients.

**Table 1:** information of 6 cases.

Item	Gender	Age	Principal symptoms	Diagnosis	Bosniak classification	Tumor location	The longest diameter of the tumor	Surgical approach
1	Male	45	Discovered during physical examination	Simple renal cyst of the left kidney.	IIF	The top part of the left kidney.	11.3cm	Laparoscopic decortication of simple renal cyst converted to partial nephrectomy
2	Male	14	Lumbago	Renal space - occupying lesion (left kidney)	IV	The superior pole of the left kidney	4.5cm	Conversion from partial nephrectomy to radical nephrectomy
3	Female	42	Discovered during physical examination	Renal space - occupying lesion (right kidney)	–	The superior pole of the right kidney	3.2cm	Partial Nephrectomy
4	Female	43	Pain in the abdomen	Left Renal Cyst	II–III	The top part of the left kidney	10.2cm	Partial Nephrectomy
5	Female	26	Pain in the left lumbar region	Renal Space - Occupying Lesion (Left Kidney)	III	The inferior pole of the left kidney	6.1cm	Partial Nephrectomy
6	Female	28	Painless gross hematuria	Tumor of the left kidney	–	The inferior pole of the right kidney	7.5cm	Intraoperative frozen biopsy combined with radical nephroureterectomy for carcinoma of the renal pelvis

**Figure 1:**
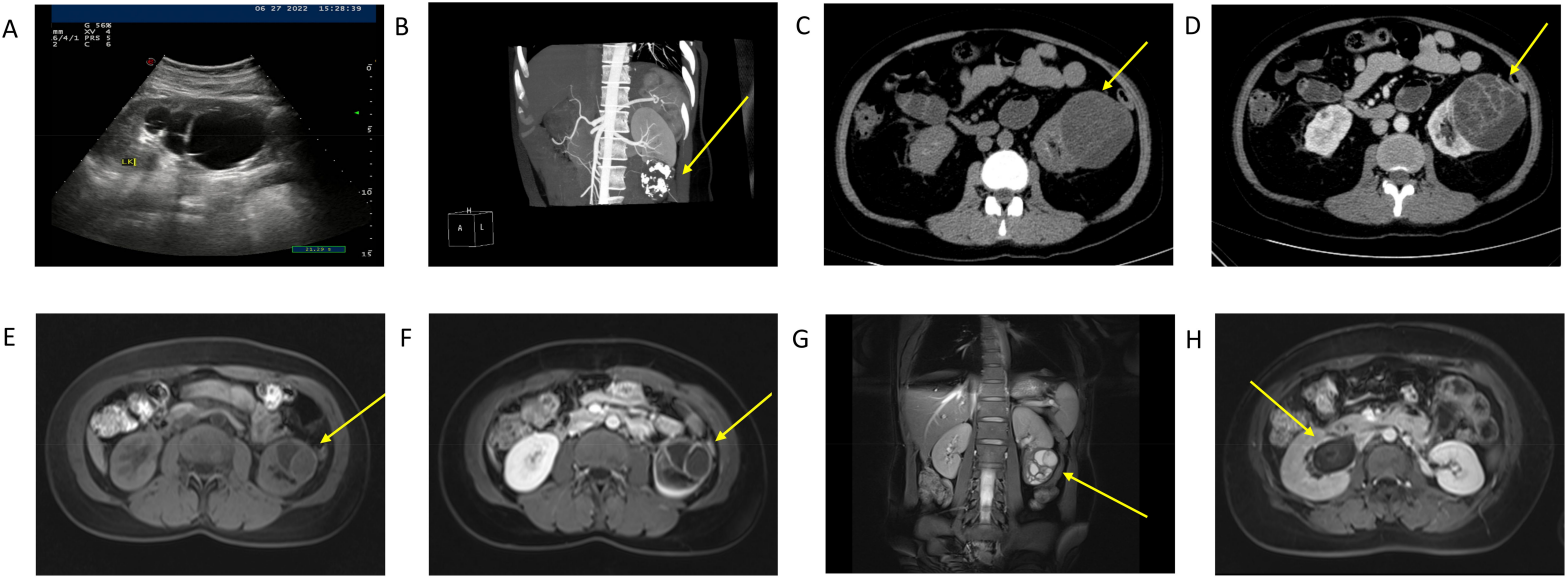
Radiological images: **(A)** (case 1): color doppler ultrasonography of the kidney. The ultrasound reveals liquid anechoic structures within the renal parenchyma, with slightly thick cyst walls and irregular grid-like septations observed inside the cysts; **(B)** (case 5): Renal artery computed tomography angiography (CTA) showing the mass at the lower pole of the kidney. Enhanced scanning of the renal artery reveals punctate enhancement within the mass; **(C, D)** (case 4): Computed tomography plain scan and contrast-enhanced imaging of cystic nephroma. Plain CT scans indicate that single oval-shaped low-density lesions are present within the kidneys. **(E–G)** (case 5): T1-weighted magnetic resonance imaging (MRI), T1-weighted contrast-enhanced MRI, and T2-weighted MRI of cystic nephroma. MRI examination shows that the cystic lesions exhibit low signal intensity on T1-weighted images and high signal intensity on T2-weighted images. Some patients present linear septations with intermediate to low signal intensity on T1-weighted images. Upon contrast enhancement, linear septations or capsular enhancements are visible; **(H)** (case 6): T1-weighted contrast-enhanced MRI demonstrating tumor-induced compression of the renal pelvis and calyces.

**Figure 2:**
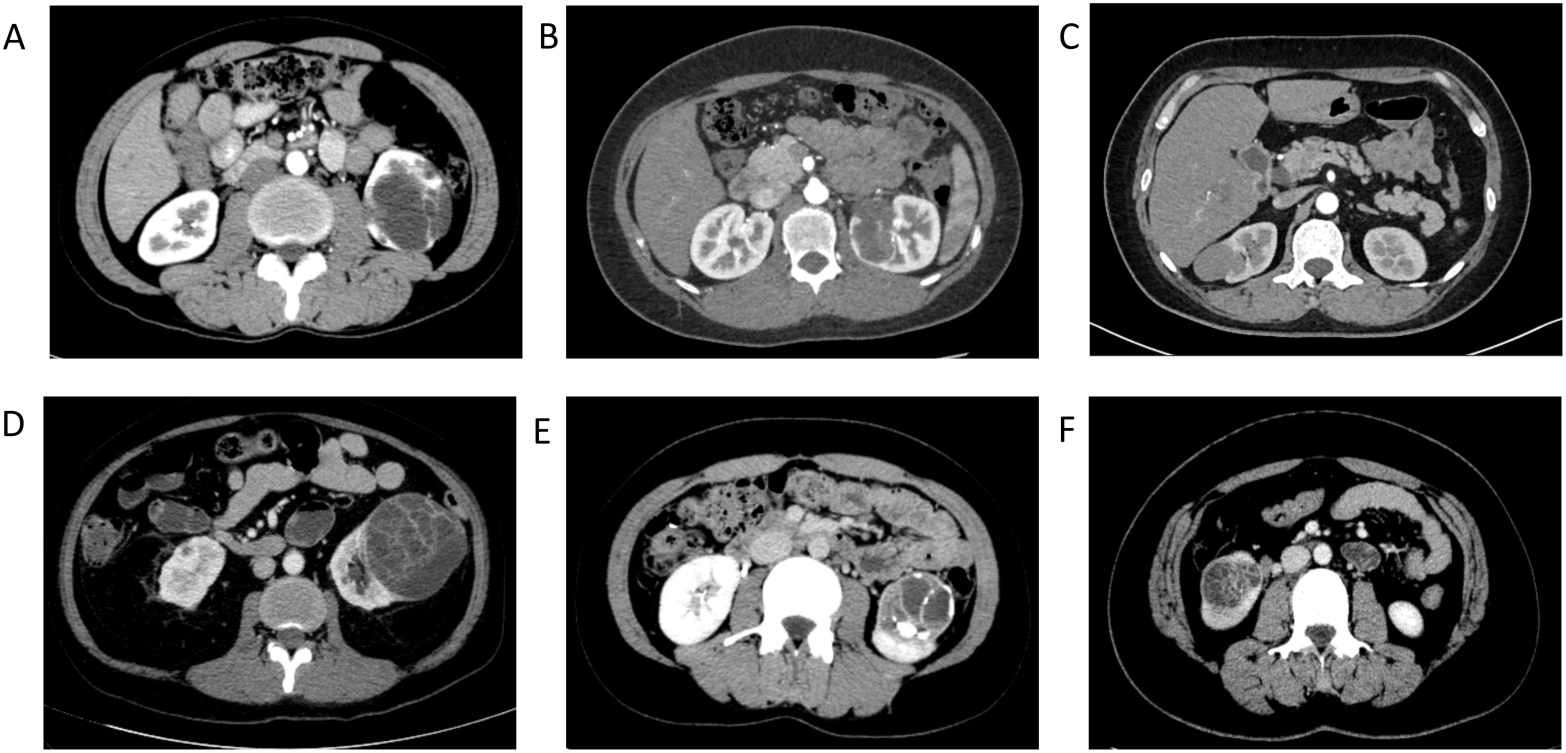
[Contrast-enhanced CT of the kidneys; **(A–F)** correspond to cases 1–6]: CT scans indicate that single or multiple oval-shaped low-density lesions are present within the kidneys; in 4 patients **(A, D–F)**, septum-like high-density shadows are visible within the lesions, and upon enhancement, the linear septa show significant enhancement;.

All 6 patients undergo magnetic resonance imaging (MRI) and computed tomography (CT) (enhanced) examinations prior to surgery. Two patients also receive color Doppler ultrasound examinations. The ultrasound reveals liquid anechoic structures within the renal parenchyma, with slightly thick cyst walls and irregular grid-like septations observed inside the cysts (**Figure 1A**). CT scans indicate that single or multiple oval-shaped low-density lesions are present within the kidneys(**Figure 1C, D**) & (**Figure 2**). In 4 patients, septum-like high-density shadows are visible within the lesions, and upon enhancement, the linear septa show significant enhancement(**Figure 2A, D, E, F**). Enhanced scanning of the renal artery reveals punctate enhancement within the mass (**Figure 1B**). MRI examination shows that the cystic lesions exhibit low signal intensity on T1-weighted images and high signal intensity on T2-weighted images. Some patients present linear septations with intermediate to low signal intensity on T1-weighted images. Upon contrast enhancement, linear septations or capsular enhancements are visible (**Figure 1E, F, G**). In a few cases, the renal pelvis and ureter may be involved. One of these cases involved a large tumor, exerting pressure on the renal pelvis and calyces and invading the upper segment of the ureter (**Figure 1H**).

**Figure 3:**
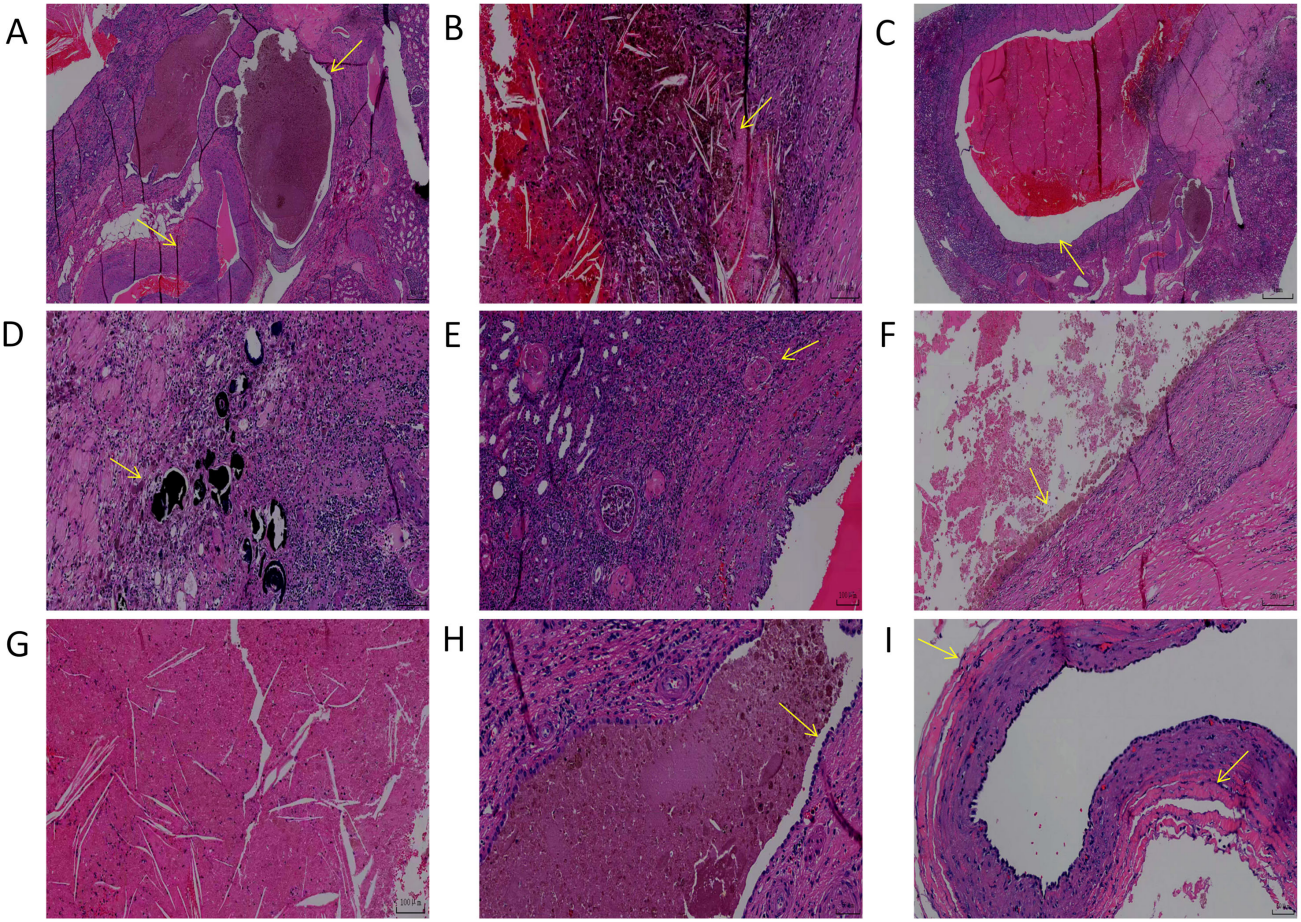
Atlas of pathology: **(A)** the tumor comprised cysts of varying sizes within the renal parenchyma. Hematogenous materials were present within the cysts, and thick-walled blood vessels were observed in the surrounding area. **(B)** Hemorrhagic regions and hemosiderin deposits were evident in the interstitial tissue of the cysts. **(C)** Low-power magnification showed cysts of variable sizes with small amounts of pale red material in some cystic chambers. **(D)** Calcified regions and hemosiderin-laden macrophages were present within the tumor stroma. **(E)** Chronic inflammatory cell infiltration was observed in the renal parenchyma adjacent to the tumor, with some glomeruli exhibiting hyaline degeneration. **(F)** A portion of the tumor epithelium underwent necrosis and exfoliation, with necrotic material within the cystic lumen and hemosiderin deposits in the epithelial region. **(G)** Necrotic debris and cholesterol crystals were detected within the cystic cavity. **(H)** The cystic epithelium was lined by cuboidal or hobnail cells with eosinophilic cytoplasm. No cellular atypia or mitotic figures were observed. **(I)** The tumor stroma consisted of sclerotic collagenous fibers and mucinous regions, resembling ovarian-like stroma.

**Figure 4:**
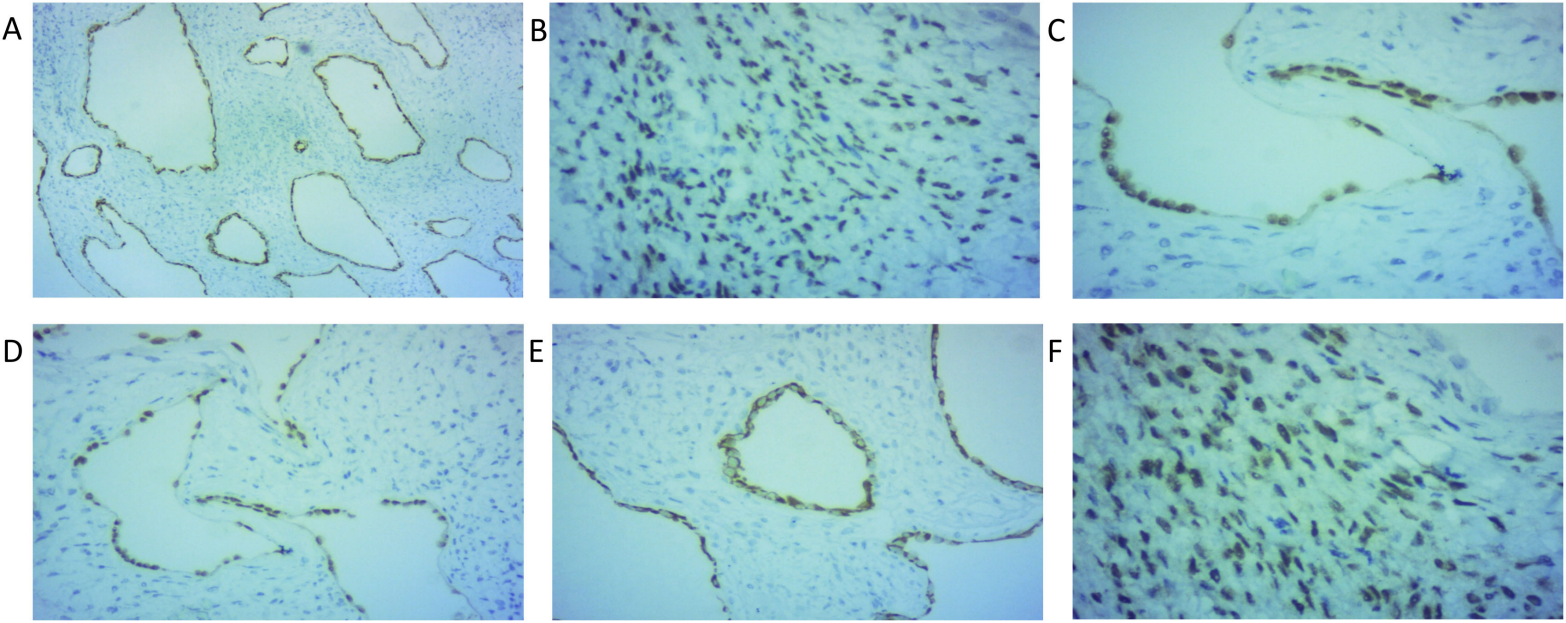
IHC results 1-marked positive: **(A)** cytokeratin CK-L 40X; **(B)** ER 200X; **(C)** PAX-8 200X; **(D)** PAX-8 100X; **(E)** cytokeratin CK-L 100X; **(F)** PR 200X.

**Figure 5:**
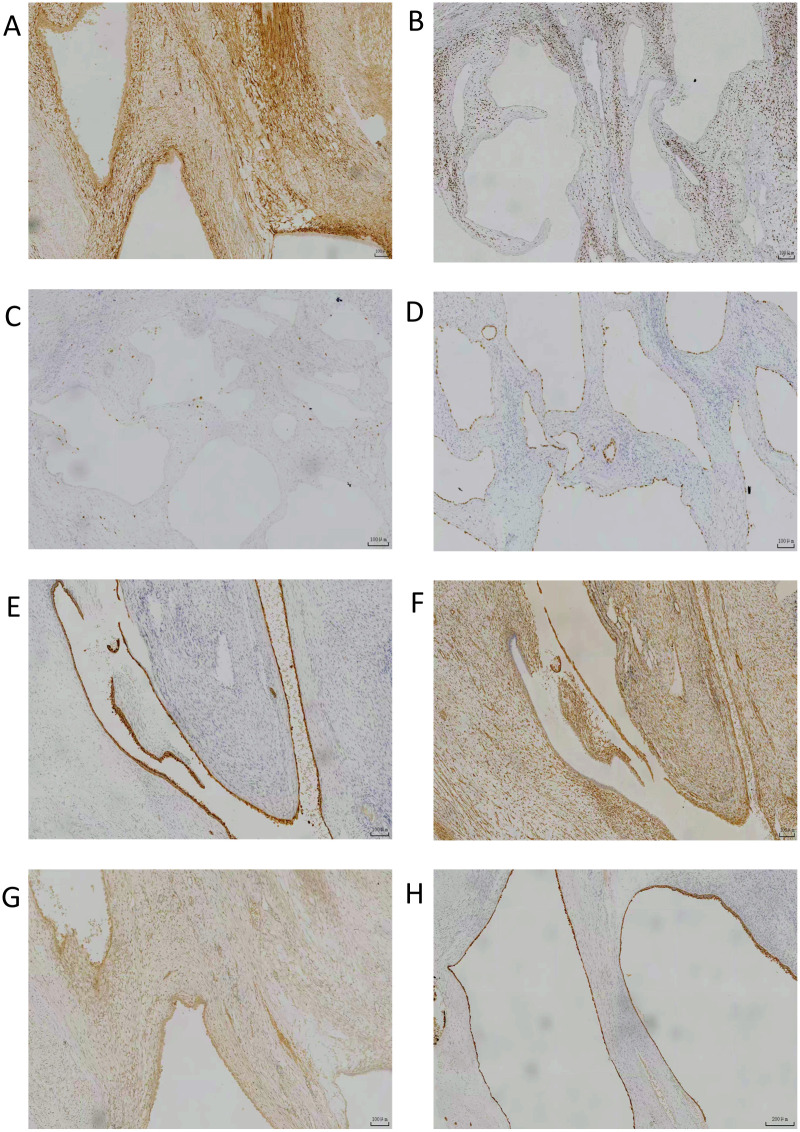
IHC results 2-marked positive: **(A)** CD10; **(B)** ER (100X);C. Ki67 (1%); **(D)** PAX-8; **(E)** CK7; **(F)** vimentin; **(G)** Inhibin-A; [**(A–G)**:40X] **(H)** CK5/6 50X.

Preoperative diagnoses included 2 cases of renal cysts, 3 cases of renal space-occupying lesions, and one case of a renal tumor. All 6 patients underwent surgical treatment. In one case, due to preoperative misdiagnosis as a Bosniak II F grade renal cyst, laparoscopic decortication of a simple renal cyst was performed. During the operation, the cyst was found to be septated, and considering the possibility of a CN, the procedure was converted to partial nephrectomy. In another case, the preoperative diagnosis was a renal tumor. Intraoperative frozen section examination revealed atypical hyperplasia and invasion of the renal pelvis, calyces, and the upper segment of the ureter. Therefore, radical nephroureterectomy for renal pelvic cancer was performed to completely resect the affected kidney and ureter. Three patients underwent partial nephrectomy. Among them, 2 cases were classified as Bosniak III grade, whereas the Bosniak grade was not provided for one case. In one instance, due to the relatively large tumor detected during the operation, the surgical approach was changed to radical nephrectomy, and this case was classified as Bosniak IV grade. All patients underwent complete histopathological examination postoperatively(**Figure 3**, **Figure 4**, **Figure 5**).

Gene testing was not performed on all 6 patients due to limitations in hospital facilities. Four patients were successfully followed up at 18, 30, 44, and 103 months post-surgery. Some patients underwent follow-up examinations at local hospitals, with three cases showing no signs of recurrence on postoperative CT scans at outpatient clinics. One patient underwent CT scanning during a follow-up at a county-level hospital. The outpatient physician noted that the renal mass had not been completely excised following the partial nephrectomy. However, during telephone follow-up, the patient reported no postoperative discomfort.

## Discussion

CN was first reported in 1892. It is a rare benign renal tumor with an unknown etiology (5, 6). In the past, cystic nephroma (CN) and mixed epithelial and stromal tumor (MEST) of the kidney were considered two similar tumor subtypes (7). Well circumscribed, multicystic tumors with no apparent solid component. Versus MEST - variable solid and cystic components. In the latest World Health Organization (WHO) classification of tumors of the urinary system and male genital organs, adult cystic nephroma (CN) is categorized as Mixed epithelial and stromal tumor of the kidney (MEST), while pediatric cystic nephroma is classified separately (8), due to the presence of a unique DICER1 gene mutation in pediatric cystic nephroma (9–13). Recent studies investigating the DICER1 gene status in adult MEST/CN cases have not identified relevant mutations. Morphological research also suggests that PCN and MEST/CN should be distinguished from one another (14–16). Statistics indicate that cystic nephroma (CN) is more common in male newborns (with a male-to-female ratio of 3:1), while its incidence is higher in middle-aged women (with a male-to-female ratio of 1:9) (17). Among the 6 patients discussed in this study, the male-to-female ratio for neonatal onset was 1:0, while for middle-aged onset it was 1:4, this is broadly consistent with previous reports (17). DICER1 tumor predisposition syndrome is a rare autosomal dominant hereditary familial tumor susceptibility disorder characterized by heterozygous DICER1 germline mutations. The most common tumor clinically is pleuropulmonary blastoma, and CN is also one of them (18). Previously reported cases describe the concurrence of pleuropulmonary blastoma and CN (10, 19). In van Peer, et al., 10 of the 12 patients with germline DICER1 mutated CN who underwent genetic testing had previous or subsequent tumors other than CN. Of the remaining 155 patients, including 9 patients with bilateral CN, germline genetic testing (DICER1 or other tests) was not described. Before and after the diagnosis of CN, a total of 26 patients developed DICER1-related tumors (such as PPB, multinodular goiter, embryonal rhabdomyosarcoma, malignant teratoid ciliary medullary epithelioma, etc.). Of the 26 patients with CN who developed other tumors, 4 developed these tumors before CN, 14 concomitant, and 8 patients developed other tumors after CN (3). No recurrences or progression to other tumor types were observed among the patients we followed up.

### Imaging of CN and reasons for misdiagnosis

Based on the 6 cases analyzed in this study, we Summarized specific imaging characteristics of CN: The ultrasound reveals liquid anechoic structures within the renal parenchyma, with slightly thick cyst walls and irregular grid-like septations observed inside the cysts. Plain CT scans indicate that single or multiple oval-shaped low-density lesions are present within the kidneys. In some patients, septum-like high-density shadows are visible within the lesions, and upon enhancement, the linear septa show significant enhancement (8, 20). MRI examination shows that the cystic lesions exhibit low signal intensity on T1-weighted images and high signal intensity on T2-weighted images (21–23). Some patients present linear septations with intermediate to low signal intensity on T1-weighted images. Upon contrast enhancement, linear septations or capsular enhancements are visible. This has rarely been reported in previous studies; a preliminary diagnosis of a malignant tumor involving the renal pelvis and kidney was made prior to surgery.

The imaging manifestations of CN often overlap with those of simple renal cysts, particularly in Bosniak Class I and Class II. The imaging characteristics, such as thin cyst walls and the absence of obvious septa, predispose it to misdiagnosis as a simple renal cyst. In some instances, preoperative imaging suggested a simple cystic tumor, but during the operation, it was revealed that the cystic cavity contained multiple septal structures, indicating an increased complexity of the lesion and possibly categorizing it as Bosniak Class III or Class IV. In this report, 2 medical records were diagnosed as simple renal cysts. One patient underwent unnecessary laparoscopic decortication of a simple renal cyst. During the operation, septated cystic cavities were found, which are characteristic of CN. Suspecting a complex cystic tumor, the surgical approach was changed to partial nephrectomy. One patient was preoperatively diagnosed with a renal tumor and underwent standardized intraoperative frozen section analysis followed by radical surgery. The remaining three cases presented with renal space-occupying lesions without definitive preoperative characterization. Our literature review revealed that clinicians tend to diagnose cases with poorly defined septal walls as renal cysts, whereas those exhibiting disorganized septations and larger lesion volumes are more frequently suspected to be renal cell carcinoma (1). Existing case reports predominantly focus on single-case descriptions, and current retrospective reviews primarily emphasize postoperative pathological findings rather than preoperative diagnostic challenges. The exact preoperative misdiagnosis rates of CN remain undetermined in published studies (17). Recent reports indicate that cystic Wilms’ tumor and CN remain difficult to distinguish on imaging (24). There have been reports of larger tumor masses (16 cm) in pediatric CN patients (22). In our report, 4/6 of cases demonstrated indeterminate benign or malignant characteristics preoperatively, with 33.3% misdiagnosed as renal cysts. In the study by Edney et al., it was mentioned that variations in the interpretation of cystic renal masses by the Bosniak system arise from multiple aspects. The latest version of the Bosniak classification of cystic renal masses provides more specific imaging diagnostic strategies and highlights the role of MRI in differentiating the benign and malignant nature of renal cystic masses (25, 26). The research by Carmen et al. proposed novel diagnostic criteria for distinguishing benign from malignant tumors by defining new radiological patterns to enhance the classification of Bosniak III and IV cystic renal masses (21). In some cases of this study, although they were diagnosed as Bosniak Class II before surgery, CN was confirmed by postoperative pathology, further illustrating the uncertainty of imaging examinations in certain complex cases. There are many potential side effects of misdiagnosed cystic nephroma.(1). Misdiagnosis may lead to incorrect surgical decisions, Laparoscopic Decortication is unnecessary for CN patients;(2).Erroneous surgical intervention resulted in incomplete eradication of the pathological focus, which carries a risk of recurrence; (3). Due to incorrect preoperative diagnosis, surgical modification may exacerbate postoperative morbidity and impose incremental healthcare expenditures (27).

### Surgical treatment strategies for CN

Adult/pediatric cystic nephroma (CN) typically requires surgical intervention, such as partial nephrectomy (e.g., for small CN cases and patients with congenital solitary kidney) or total nephrectomy. Intraoperative examination revealed that most cases exhibited a mass protruding from the renal capsule. In some cases, complex multilocular cystic masses were visible on the renal surface. The tumors were primarily located within the renal parenchyma. One case showed tumor involvement of the lower calyx of the renal pelvis, suggesting potential malignant potential, with similar cases documented previously (28). CN is often misdiagnosed as a simple renal cyst, leading to unnecessary surgical treatments, such as laparoscopic decortication of simple renal cysts. Attempted laparoscopic decortication in the setting of multiple complex cystic cavities may affect prognosis. Postoperative tumor recurrence may correlate with incomplete tumor resection (2).

Early partial nephrectomy proves more beneficial for prognosis in cases with ambiguous imaging classified as Bosniak Class I-II. Some researchers emphasize the significance of preoperative percutaneous puncture to determine the benign or malignant nature of the mass. However, the risk of tumor cell dissemination resulting from puncture must also be considered (17). For example, an earlier review included 179 patients with multilocular cystic nephroma (the term used prior to the new classification), spanning the period from 1892 to August 2014. Among these, 135 underwent radical nephrectomy, 31 underwent partial nephrectomy, and 1 received percutaneous aspiration or puncture therapy. Follow-up revealed 6 cases of malignant transformation (17). In a literature review on pediatric cystic nephroma (CN) and Wilms tumor, among 167 CN patients included in the study, one case of local recurrence was reported during post-treatment follow-up, potentially due to incomplete tumor resection. Six patients died during follow-up: one from cerebral hemorrhage during surgery for brain spindle cell sarcoma, and five from complications during treatment for concomitant pleuropulmonary blastoma (PPB) (3). None of the patients included in this report had a history of percutaneous aspiration or puncture procedures, and no cases of disease progression were observed during follow-up.

### Pathology and differential diagnosis of CN

Postoperative pathological diagnosis remains the gold standard for the definitive diagnosis of CN. CN cysts are: Noncommunicating cysts lined by single layer epithelium separated by septa with hypocellular fibrous to hypercellular spindle cell stroma (13). Postoperatively, the gross specimens obtained were mostly multilocular cystic masses with capsules. Upon incising the local area, multiple septated cystic cavities were visible, predominantly grayish-white or grayish-red in color. Light yellow or clear fluid flowed from the cavities, whereas in some patients, the cystic fluid appeared coffee-colored or accompanied by bleeding. Microscopic findings (**Figure 3****):** Low-power microscopy revealed a tumor composed of cysts of varying sizes within the renal parenchyma. Hematogenous materials were present within the cysts, and thick-walled blood vessels were observed in the surrounding area (**Figure 3A-C**). The cyst epithelium was lined with cuboidal or hobnail-like cells with eosinophilic cytoplasm (**Figure 3H**). Small clusters of polygonal cells with amphophilic cytoplasm and round nuclei, frequently around epithelial component (11). Degenerative and necrotic changes, including hyaline degeneration and foamy or hemosiderin laden macrophages, focal chronic inflammation and Calcifications were noted in the cyst stroma. No cellular atypia or mitotic figures were identified. In some regions, the tumor epithelium was necrotic and sloughed off, (**Figure 3D, E, F, G**). Examination of the cyst fluid detected a few tumor cells and histiocytes. The stroma within the septa between the cystic cavities represents a notable cellular characteristic of this disease. The septa consist of hypocellular fibrous connective tissue. The tumor stroma contains sclerotic collagen fibers and mucinous regions, collagenous and fibrous to edematous and myxoid, with some areas resembling ovarian-like stroma (**Figure 3I****) (**29). In the three cases where Ki-67 IHC examination was conducted, the results were consistently around 1%, indicating that the tumor is classified as benign. The five pathological diagnostic criteria for CN proposed by Boggs et al. include:

a) Presence of a multilocular cystic lesion.
b) Majority of cystic cavities lined by epithelial cells.
c) Absence of communication between cystic cavities and the renal pelvis.
d) Residual renal tissue outside the tumor capsule with preserved architecture.
e) Absence of immature renal elements within septal stroma (30).

In our reported cases, the postoperative pathological findings were fully consistent with these essential diagnostic criteria.

Among these 6 patients, 4 underwent immunohistochemical (IHC) testing: Cells expressing cytokeratin (CK), representing epithelial and mesenchymal origins, and smooth muscle actin (SMA) were positive. Estrogen receptor (ER) and progesterone receptor (PR) staining were typically positive (2). Cystic neoplasms (CN) predominantly affect women aged 20–50 years. Hormonal fluctuations within this age group—such as those occurring around menopause—may contribute to the development of cystic neoplasms. Recent studies indicate that estrogen receptor (ER) expression in DICER1-related lesions correlates with the presence of cystic components (31). All four cases demonstrated PAX-8 positivity. Recent studies have revealed the critical role of PAX-8 in the differentiation of renal mesenchymal cells into epithelial cells (32). PAX8 transcription factor is essential for the survival of differentiated epithelial cells. Recent evidence indicates that the PAX8 gene is overexpressed in most epithelial-derived renal tumors, providing strong molecular evidence that CN is a tumor with dual epithelial and mesenchymal cell origins (33). Other low-positive markers include CK7, CD10, vimentin, and other immunological indicators. Studies indicate that most (83%) adult cases show at least focal staining for inhibin, while no pediatric cases exhibit inhibin staining (**Figures 4**, **5**; **Table 2**) (15). The study concludes that the pathological differences between adult and pediatric cystic nephromas lie in the fact that adult cystic nephromas typically contain ropy collagen and stain for inhibin (8).

**Table 2:** IHC results of 4 cases.

Item	PR	ER	PAX-8	CK	SMA	CD10	Vimentin	CK7	WT-1	Inhibin-A	Ki-67	CK5/6	CKL	MC	CD117
1	+	+	+	+	+	_			_	_					
2			+	+		_		+			<1%		+		_
3	+	+	+	+					_		1%	+	+	+	
4	_	+	+		+	+	+			+	1%				+

Cystic renal cell carcinoma (CRCC) is the most common malignant cystic tumor. Its pathological manifestation is clear cell carcinoma with cystic degeneration, which shows significant histopathological differences from CN (34). Renal mucinous cystadenoma belongs to renal malignant tumors and is extremely rare clinically. It is a neoplasm that may present as a single capsule or a multilocular tumor containing jelly-like mucus. During microscopic examination, a single layer of high columnar mucinous epithelium can be found within the wall, which is distinctly different from CN (35). Through a detailed analysis of the tumor tissue, a more accurate distinction between benign and malignant lesions can be made, thereby providing a basis for postoperative treatment and follow-up. In the present case described above, only one patient underwent an intraoperative frozen section biopsy, which confirmed the diagnosis of CN, and a radical pyelectomy was performed. For the remaining five cases, the pathological diagnosis was determined only after postoperative pathological examination. Our retrospective analysis of IHC profiles in multilocular cystic renal cell carcinoma (MCRCC) and clear cell RCC (ccRCC) revealed the following differential expression patterns:

CD10: 63% positivity in MCRCC vs. 96% in ccRCC.

CK7: 92% vs. 38%.

α-methylacyl-CoA-racemase (AMACR): 21% vs. 67%.

Vimentin: 58% vs. 33%.

Estrogen Receptor (ER): 8% vs. 8%.

CAM 5.2: 100% vs. 96%.

EMA, CA-IX, PAX-2: Universal positivity (100%) in both groups (36).

Distinct hormonal receptor profiles were observed between the renal tumor subtypes: neither MCRCC nor ccRCC exhibited significant ER or PR expression (positivity rates <5% for both markers), whereas CN demonstrated frequent hormone receptor positivity.

The Ki-67 proliferation index further stratifies diagnostic categories; histologically aggressive tumors, such as MCRCC and ccRCC, exhibit elevated indices that correlate with nuclear grade progression. This proliferative gradient (RCCs >10% vs. CN <5%) aligns with the 2022 WHO classification criteria for renal tumor biological behavior, where Ki-67 ≥15% serves as a validated threshold for predicting metastatic potential (13, 37). Similarly, we reviewed the characteristics of CPDN (Cystic partially differentiated nephroblastoma) as well as cystic nephroblastoma and other genetically related polycystic kidney diseases, and a retrospective study by Sophie E van Peer et al. noted that In children with predominantly cystic renal tumors, the most likely diagnoses include cystic partially differentiated Wilms tumor (CPDN) and cystic nephroma (CN). They noted that children with CPDN and CN were younger and had no metastases, and surgery was the primary treatment for both. And the prognosis is good, which is consistent with the cases we have reported in children (3).

CPDN has no specific clinical features, most patients only show painless abdominal masses found by accident or renal tumors found by abdominal ultrasound or CT, similar to the clinical features of CN, Liu et al. summarized the pathological characteristics of CPDN based on previous reports: The cyst is lined by a flattened, hobnail, or cuboidal epithelium. The septa may contain epithelial structures resembling mature renal tubules and may also contain blastema, with or without embryonic stromal or epithelial elements (38). In addition, the various neoplastic conditions like ACD-associated RCC, cystic papillary renal cell carcinoma, tubulocystic carcinoma, regressing clear cell renal cell carcinoma (ccRCC) with cystic degeneration should be kept as histopathological differential diagnoses.

Research by PDQ Cancer Information Summaries and Andrew M Fleming et al. states that Wilms tumor is the most common primary kidney malignancy in children, with an average age of diagnosis of unilateral cases of Wilms at 44 months, or about 10% The children with Wilms tumor have congenital anomalies. Wilms tumors often manifest as abdominal masses and should be differentiated. About one-third of Wilms tumor cases involve variants in WT1, CTNNB1, or AMER1 (WTX). Autosomal dominant polycystic kidney disease is the most common monogenic hereditary kidney disease. PKD1 and PKD2 are the most common mutant genes in the pathogenesis of ADPKD. These patients are at high risk of developing accelerated renal failure after surgery and require regular monitoring of treatable manifestations of ADPKD. When ADPKD is diagnosed preoperatively, a comprehensive preoperative evaluation of the cystic kidney disease in children is necessary, at which point the primary renal tumor can be evaluated simultaneously with MRI and the renal cyst can be identified (39, 40). In addition, recently reported cases of anaplastic sarcoma of the kidney (ASK)—a multicystic tumor with a solid component—suggest that CN should also be differentiated from other renal tumors in DICER1 syndrome (41). CD10, CK7, CAM 5.2, EMA, CA-IX, and PAX-2 are predominantly positive, although their expressions are rarely detected in CN. A commonality among these tumor types is the expression of SMA and vimentin, which may be associated with the involvement of epithelial-derived cells in the development of these tumors, They are involved in many tumors (42, 43). The limitations of this report include the absence of genetic testing and the small number of cases. Given the limited number of case reports available, large-scale prospective studies remain to be conducted.

### Prognosis

CN-Most benign but very rare local recurrence and malignant transformation (44). As mentioned earlier, none of the four patients successfully followed up externally showed any clear signs of recurrence. Previous studies have reported no signs of recurrence in pediatric CN patients following a 10-year postoperative follow-up, indicating a favorable prognosis with appropriate treatment (45).

## Conclusion

CN (Adult and Pediatric cystic nephroma) is a rare benign tumor that is easily misdiagnosed. Imaging examinations, particularly the Bosniak classification system, and intraoperative frozen pathological examinations are crucial for diagnosing CN. We recommend genetic testing, The benefits of DICER1 testing is that a diagnosis of DICER1-related tumor predisposition can be established and other family members can be identified so surveillance can be started, in suspected pediatric CN cases to confirm diagnoses through genetic screening, thereby guiding the selection of appropriate surgical strategies. To decide about the best treatment one should include each of these patients in an institutional interdisciplinary tumor board meeting to gain the best results and outcome for the patients. For Bosniak Class III and IV, as well as Class I-II cystic renal masses of unknown lesion nature and cases involving renal pelvis invasion, active surgical intervention and postoperative follow-up are particularly important. We recommend personalized surgical treatment based on patient status: performing radical surgery in patients with massive tumor burden, partial nephrectomy in those eligible for nephron-sparing procedures, and long-term postoperative follow-up for patients with congenital solitary kidney. In cases of high-grade CN, we typically consider giant CN masses or CN invading the renal pelvis, one of which is included in this report. We recommend radical surgery when renal function remains unaffected. Intraoperative frozen section examination combined with partial nephrectomy, can prevent incomplete tumor resection during the operation and reduce the risk of tumor recurrence. As a supplementary part of pathological examination, Immunohistochemical examination is helpful for assessing tumor invasiveness, thereby enabling the selection of a reasonable subsequent treatment plan and the determination of the postoperative follow-up cycle.

## Data Availability

The original contributions presented in the study are included in the article/supplementary material. Further inquiries can be directed to the corresponding authors.
